# Are we ready for scaling up restoration actions? An insight from Mediterranean macroalgal canopies

**DOI:** 10.1371/journal.pone.0224477

**Published:** 2019-10-25

**Authors:** Laura Tamburello, Loredana Papa, Giuseppe Guarnieri, Laura Basconi, Serena Zampardi, Maria Beatrice Scipione, Antonio Terlizzi, Valerio Zupo, Simonetta Fraschetti

**Affiliations:** 1 CoNISMa, Roma, Italy; 2 Stazione Zoologica Anton Dohrn, Napoli, Italy; 3 Department of Biology, and Environmental Sciences and Technologies, University of Salento, Lecce, Italy; 4 Department of Environmental Sciences, Informatics and Statistics, Ca’ Foscari University, Venice, Italy; 5 Department of Life Sciences, University of Trieste, Trieste, Italy; 6 Department of Biology, University of Naples Federico II, Napoli, Italy; University of Barcelona, SPAIN

## Abstract

Extensive loss of macroalgal forests advocates for large-scale restoration interventions, to compensate habitat degradation and recover the associated ecological functions and services. Yet, restoration attempts have generally been limited to small spatial extensions, with the principal aim of developing efficient restoration techniques. Here, the success of outplanting *Cystoseira amentacea* v. *stricta* germlings cultured in aquaria was experimentally explored at a scale of tens of kms, by means of a multifactorial experimental design. In the intertidal rocky shores of SE Italy, locations with a continuous distribution for hundreds of meters or with few thalli forming patches of few centimeters of *C*. *amentacea* canopy were selected. In each location, the effects of adult conspecifics and the exclusion of macrograzers (salema fish and sea urchins) on the survival of germlings were tested. We evaluated the most critical determinants of mortality for germlings, including the overlooked pressure of mesograzers (e.g. amphipods, small mollusks, polychaetes). Despite the high mortality observed during outplanting and early settlement stages, survival of *C*. *amentacea* germlings was consistently favored by the exclusion of macrograzers, while the presence of adult conspecifics had no effects. In addition, the cost analysis of the interventions showed the feasibility of the *ex-situ* method, representing an essential tool for preserving *Cystoseira* forests. Large scale restoration is possible but requires baseline information with an in-depth knowledge of the species ecology and of the areas to be restored, together with the development of specific cultivation protocols to make consistently efficient restoration interventions.

## Introduction

Coastal ecosystems are globally threatened by multiple and interacting anthropogenic stressors [1]. Shifts from high diversity systems to low diversity ones have been frequently observed, and degraded conditions are often irreversible unless external interventions force the natural recovery of ecosystems [2]. Efforts aimed at mitigating human impacts through management of human activities and conservation of biodiversity, in the attempt of reverting present trajectories of changes, are not always successful [3], and natural recovery of degraded marine ecosystems rarely occurs in absence of specific conservation strategies [4]. In this scenario, active restoration, alone or in combination with other forms of regulations (e.g. Marine Protected Areas), is considered an effective strategy to assist and speed up the recovery of degraded ecosystems or to foster the backward shift from degraded states [5, 6].

Macroalgal forests (including fucoids and kelps) are severely threatened by several stressors such as direct degradation or destruction of habitat, coastal urbanization, pollution, and herbivores outbreaks, acting in combination with climate change [7–9]. Their decline over vast extensions has been documented in many temperate regions, including the North and South Eastern Pacific [10, 11], the North Eastern and Western Atlantic [12, 13], and the Australian coasts [14, 15]. As many other habitat-forming macroalgal species worldwide, *Cystoseira* forests have been declining in the whole Mediterranean basin during the last decades [16, 17], and natural recovery has been recorded only occasionally [18, 19]. Although preserving such complex and highly productive habitats represents a priority to maintain the associated biodiversity and ecosystem functioning, restoration of macroalgal forests has been largely neglected compared to other marine habitats [20, 21].

Different methodologies have been attempted for macroalgal reforestation, most frequently testing the efficacy of transplanting adult or juvenile thalli [22–24]. The technique is regarded as successful for several species of Laminariales or Fucales, exhibiting survival rates close to 70% of transplanted adult individuals for *Cystoseira* species [9, 25, 26]. Yet, the majority of studies revealed spatially variable outcomes, with very low rates or no success of the method, possibly due to dislodgement by intense hydrodynamism [23, 27–29]. Most of these interventions have been carried out at small spatial scales within single locations (never exceeding a few meters) and were limited to a few tens of individuals, with the main purpose to test the efficacy of the method or to apply specific hypotheses within experimental studies (e.g. [26–30]). Yet, the fast growth rate and the high recruitment typical of several kelp species (e.g. *Phyllospora comosa* [23]), can favor the successful re-establishment of self-sustaining, expanding adult populations after small-scale interventions, with negligible impact on donor population [23]. On the contrary, the removal of large numbers of adult individuals is hardly sustainable for existing *Cystoseira* beds and, due to the scarce resilience of compromised canopies, it would result in an irreversible disturbance of donor forests [31, 32].

The outplanting of *Cystoseira* germlings cultured in laboratory conditions has been suggested as more sustainable than destructive restoration methods [25, 33]. The harvesting of a relatively limited number of fertile apices can provide great amounts of germlings available for large-scale interventions. The natural mortality during the pre- and post-settlement ontogenic shifts (i.e. zygote polarization, adhesion to substrata) can be strongly reduced and the growth to the germling phase can be enhanced through specific cultivation protocols (i.e. optimizing light and nutrient supply), avoiding grazing pressure on eggs and zygotes [30, 33]. Yet, the outplanting process and early settlement stages are likely to be critical for the survival and growth of *Cystoseira* germlings, representing potential bottlenecks for the realization of large-scale interventions.

Herbivory represents one of the major threats for the survival of *Cystoseira* juveniles once reintroduced at restoration sites, as it can drastically limit the survival of reintroduced individuals, with estimated losses up to 90% in a few days [34]. In the Mediterranean Sea, grazing by sea urchins and salema fish (*Sarpa salpa* Linnaeus, 1758) can cause drastic decline of *Cystoseira* forests, both in shallow subtidal and in intertidal rocky shores [35–37]. In the latter habitat, also limpets and other gastropods may negatively affect the survival of juveniles [38]. Limitation of grazers pressure through caging may represent an efficient strategy to enhance survival of juveniles in the early stages of settlement [28]. However, small-sized recruits can be severely affected also by grazing or bull-dozing disturbance due to mesograzers (e.g. amphipods, small crustaceans and gastropods, polychaetes; *sensu* Duffy and Hay [39]), which can be hardly prevented and whose effects are unknown. In addition, the presence of adult *Cystoseira* conspecifics might ameliorate abiotic conditions and facilitate juvenile survival, by mitigating desiccation stress [40, 41]. Yet, several studies reported lack or negative effects of canopy forests, as juvenile growth might be limited by competitive shading or whiplash [38, 40, 42, 43].

Disentangling the role of the drivers affecting a restoration intervention is a priority to increase the potential of success in reforestation, especially if the challenge is restoring *Cystoseira* at spatial scales relevant to restore its loss. We focused on *Cystoseira amentacea* v. *stricta* Montagne (hereafter *C*. *amentacea*) since the genus is considered “of community interest” according to the EU Habitat Directive (92/43/EEC). *Cystoseira amentacea* has been included among the indicators of environmental quality in Mediterranean coastal waters according to the Water Framework Directive (2000/60/EC), (i.e., EEI and CARLIT). In addition, it is among the species recognized as a priority by the Barcelona Convention and considered vulnerable by several international organizations (e.g. IUCN, RAC/SPA, MedPan), as it is particularly exposed to a suite of human threats. Its reduction/loss has been recorded in several locations in the NW Mediterranean [33, 44], including our study region. However, connectivity among populations is known to be very low, this preventing the recovery of the species once disappeared in one area [45]. Here, the potential of the outplanting of *C*. *amentacea* germlings cultured in aquaria was experimentally explored testing the approach for the first time following a hierarchical design, which included the scales of meters, hundreds of meters, and tens of kilometers, allowing to replicate the outplanting technique through space. The manipulation and deployment of tens of thousands of germlings implied facing logistic constraints during the outplanting phase, and contributed to identify the key ecological knowledge and the methodological issues to be addressed for a successful scaling up of *Cystoseira* restoration.

More specifically we examined 1- the variation in the abundance of germlings during all the outplanting steps; 2- the separate and combined effects of the presence of adult conspecifics and the exclusion of macrograzers (i.e. salema fish and sea urchins) on the abundance of *Cystoseira* recruits during early settlement phases (i.e. after three months), aiming at identifying which conditions can increase the number of recruits reaching the adult stage; 3- the efficacy of adult transplant as a driver of germlings survival. The final aim was to understand the feasibility of a restoration intervention carried out at a spatial scale of tens of kms also in terms of associated costs, quantifying the mortality associated to each step of the experiment, from the culture in the laboratory to outplanting in the field.

## Materials and methods

### Field experimental set up

**Fig 1:**
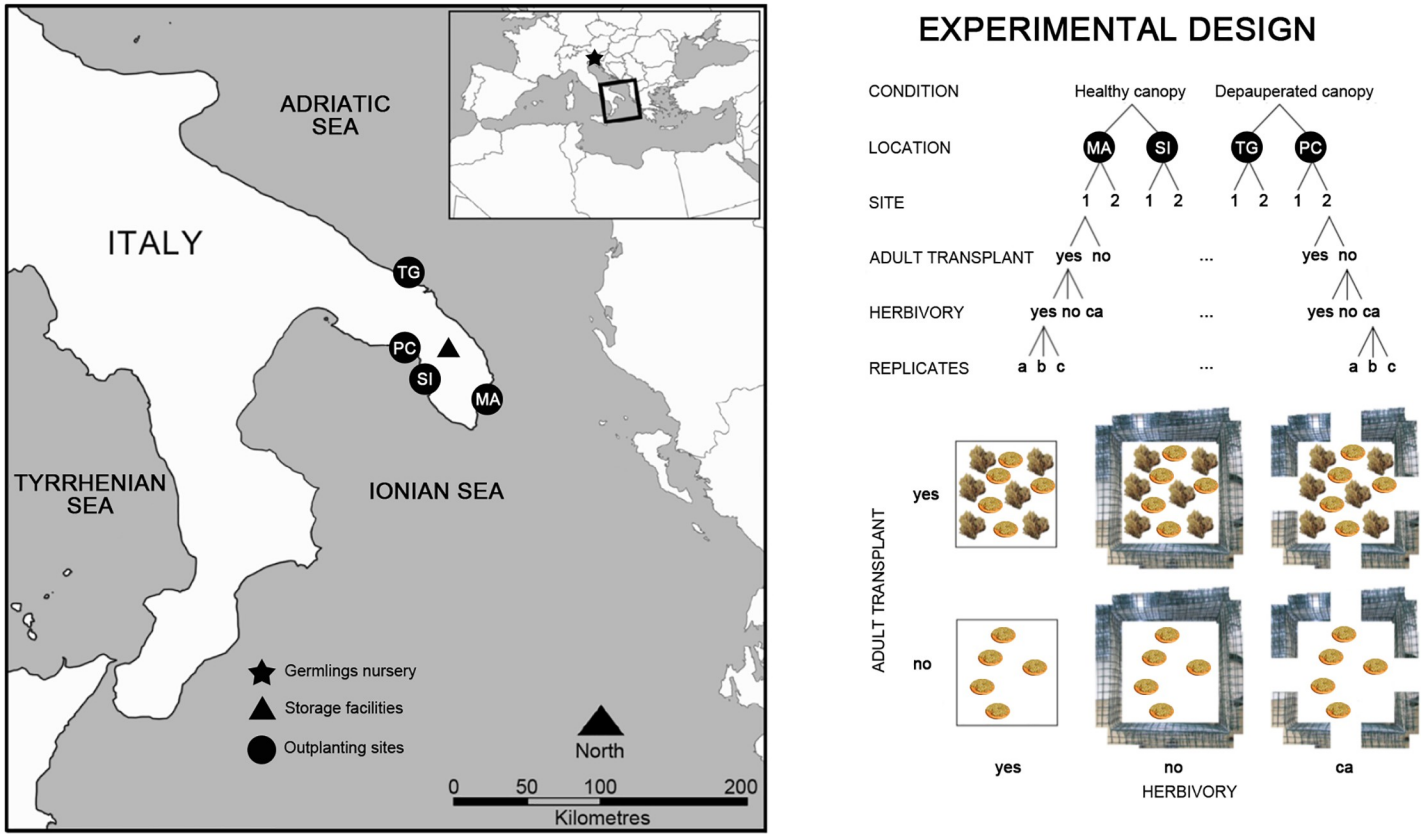
Experimental design and locations. On the left side, map of nursery (University of Trieste), storage (University of Salento), and experimental locations. MA = Marittima (Donor); SI = Sant’Isidoro (Donor); TG = Torre Guaceto (Restoration); PC = Porto Cesareo (Restoration). On the right side, the experimental design and treatments are schematically represented. CA = control of artifact. Maps were created using ArcGIS1 10.1 software by ESRI (Environmental Systems Resource Institute, www.esri.com).

In the intertidal (± 30 cm MSL) rocky shore of Apulia, SE Italy, two alternative conditions were identified: assemblages dominated by a dense canopy of *Cystoseira amentacea* (> 80% cover), or with sparse and rare individuals of *C*. *amentacea* (< 10% cover), representing eligible restoration sites. Two locations for each condition (i.e. donor sites: Marittima and Sant’Isidoro; and restoration sites: Torre Guaceto and Porto Cesareo) were randomly selected among those featured by the requested conditions at a distance of tens of kilometers (Fig 1). On the Adriatic Sea, Marittima (39.997° N, 18.419° E) was characterized by a dense canopy of *C*. *amentacea* along the vermetid trottoir with a distribution continuous for hundreds of meters, in an area with limited public access. Both Torre Guaceto and Porto Cesareo are Marine Protected Areas and do not show any evident sign of human threat able to impair this restoration intervention. Within the no-take zone of the MPA of Torre Guaceto (40.716° N, 17.799° E), a rocky platform with sparse individuals of *C*. *amentacea* was selected. The historical presence of dense canopy beds is described by Fraschetti et al. [46], but the species severely declined about ten years ago [47]. On the Ionian Sea, within the MPA of Porto Cesareo, two locations, either characterized by dense canopy beds (Sant’Isidoro; 40.193° N, 17.919° E) or by sparse individuals (Porto Cesareo; 40.247° N, 17.898° E), were identified. Guarnieri et al. [48] confirmed the historical presence of *C*. *amentacea* beds in both locations, although the species has nearly disappeared in Porto Cesareo. Paradoxically, the two locations chosen for restoration, where *Cystoseira* forests have declined, are within MPAs, both Special Protected Areas for the Mediterranean. The MPA of Torre Guaceto has been recently nominated for a Global Ocean Refuge System Award for its commitment to excellence in marine conservation. The reasons for the decline of the macroalgae are not known, but its conservation status makes the MPA as an excellent area where to implement a restoration intervention. Porto Cesareo is more embedded in a human seascape, but management is effective and water quality is considered very high in the area where the intervention was planned [48].

At all experimental sites, substrate was nearly horizontal or slightly sloping, and it was either a mixture of vermetid trottoir and rocky platform (Marittima and Sant’Isidoro) or calcarenitic rock (Porto Cesareo and Torre Guaceto). For the duration of the whole experiment, no thermal anomalies were observed in the study region. As far as nutrients, nitrate concentration ranged between 0.700 and 1.545 μmol /l, while fosfate concentration ranged between 0.005 and 0.006 μmol/l in Porto Cesareo and Torre Guaceto, respectively. More information on environmental variables and human pressures for Porto Cesareo, Torre Guaceto and S. Isidoro are available at the website ***(https://amare.interreg-med.eu/)*.**

Collections and transplantations were carried out under specific permits provided from the MPAs at Torre Guaceto (Prot. 0000995-PM-17, 21/04/2017) and Porto Cesareo (Prot. 0000745-PM-17, 10/04/2017), while no specific permits were required in Marittima, because it is not part of a protected or private area.

Within each location, two sites (tens of meters long) a few hundreds of meters apart were identified. In each site, 18 experimental units (20 x 20 cm quadrats) were permanently marked with epoxy putty and randomly assigned to different treatments.

During the season of maximum vegetative development (June—July) [45], adult thalli of *C*. *amentacea* were transplanted in half of the quadrats at restoration sites, in order to obtain a density comparable to that observed in healthy assemblages (approximately 13 clumps of thalli per quadrat). Specimens were collected from half of the quadrats at donor sites and clumps of adults were dislodged with hammer and chisel, paying attention not to damage their basis. All removed specimens were stored in refreshed coolers with seawater and transported to restoration sites, where they were glued to the substratum with portions of epoxy putty on the bases. Alluminium frames with PVC strings anchored to the substrate facilitated the attachment phase, which was completed within the same day. Specimens’ attachment could require repeated trials before being successful and detached individuals were replaced within the first weeks. Frames were subsequently removed.

As a procedural control of the technique, *C*. *amentacea* was cross-transplanted in 3 quadrats between sites of different donor locations (i.e. Marittima and Sant’Isidoro), in order to expose individuals to a similar manipulation and to estimate mortality stress due to handling of *C*. *amentacea* adults. Also, in one site for each donor location, specimens were dislodged and relocated in the same position in 3 quadrats, and 3 quadrats were translocated from one site to the other within the same location.

To evaluate the effect of macrograzers on *C*. *amentacea* transplants, 30 x 30 x 40 cm exclosure cages were placed in 12 quadrats within each site. Fences made by 0.5 cm double metal mesh were screwed to the substratum and sealed with epoxy putty. Half of the cages had 4 x 4 cm openings on each side, to allow the access of herbivores (e.g. sea urchins, sea snails) and evaluate eventual effects other than herbivory due to the presence of the cage, as control of artifact. For the same reason, 6 quadrats were unfenced in each site, to be accessible to macrograzers (a scheme of the experimental design is presented in Fig 1).

### Nursery settings

In June 2017, when *C*. *amentacea* fronds were fertile at donor sites, apices were collected for fertilization and cultivation of germlings in aquaria. Apices (3–4 cm long) were cut with scissors from about 3000 fronds from the two donor locations, collecting almost 3 fertile apices from each individual, to reduce the risk of sampling highly related specimens due to self‐recruitment, ensuring sufficient genetic variability and avoiding to compromise the reproductive capability of the specimens [33]. Apices packed in aluminum foil were wrapped with seawater-wetted towels and kept in cool, humid and dark conditions during transportation to the nursery facility, to avoid gametes spawning. Transport was completed within 30 hours from collection.

In the nursery, the temperature and photoperiod were selected to reflect typical seasonal conditions at the sampling time during the reproductive season of *C*. *amentacea* (June—July). The photoperiod was set to 15:9 h light:dark cycle and the temperature at 20° C; light was provided by LED lamps (AM366 Sicce USA Inc., Knoxville, USA), and irradiance was measured with a LI-COR LI-190/R Photometer (LICOR-Biosciences, Lincoln, NE, USA). Light irradiance was set at 125 μmol photons^−2^s^−1^ for the first three days and then at 100 μmol photons^−2^s^−1^. Stosch's enriched filtered and autoclaved seawater was used as culture medium following Falace et al. [33]. As the apices were strongly epiphyted, during the first days of culture bacteria and other epiphytes (i.e. diatoms, cianophyta) proliferated. To reduce the growth of these organisms, capable to outcompete germlings, nutrient inputs were reduced to a concentration of 50% of the Stosch's enriched seawater.

A total of 720 clay tiles (ca. 4 cm diameter) were placed in 12 aquaria. Water was continuosly areated by bubbling in the center of each aquarium, and the culture medium was totally renewed every 3 days to minimize possible limiting effect of nutrients depletion.

Once in the laboratory, fertile apices were gently cleaned with a brush and rinsed with sterile seawater, to remove the adhering biofouling and detritus on their surface. Three randomly chosen apices were placed on each clay tile, to guarantee a wide coverage of settled germlings. After 2 hours, gametes were released and were visible on the substrata, so that the apices with receptacles (i.e. swollen ends of the frond containing the reproductive structures) were removed. Cultured germlings grew on clay plates for 24 days, reaching about 0.5 cm of size, before being transported to the storage location and subsequently to the field.

### Germlings outplanting

Tiles with germlings were transported from the nursery facility to the experimental locations in July 2017. Tiles were packed to minimize physical damage and maintained in cool conditions during transport. Within 12 hours, they were stored in the laboratory in proximity to restoration sites (storage location, Fig 1), with controlled temperature (22°C). Temperature was slowly increased compared to the nursery value (20°C), in order to acclimatize germlings before outplanting them in the field, where the temperature was 25°C. Outplanting of germling tiles in the field was completed within 3 days. In the storage location, culture medium was replaced with filtered seawater, which was renewed daily. Tiles were maintained in cool conditions during transport from the storage location to the field. In each quadrat, 5 tiles with germlings were fixed with epoxy glue in the inner 20 x 20 cm.

### Experimental sampling

Before transporting germlings from nursery facilities to the storage location (i.e., day 24), 194 randomly chosen tiles from different aquaria were photographycally sampled to quantify the number of recruits. All 720 tiles were sampled in the same way before being fixed to the shore, to estimate the number of germlings that reached the experimental locations during the three days of outplanting (i.e., 180 tiles on day 26, 360 tiles on day 27, 180 tiles on day 28).

The abundance of juveniles was assessed by mean of photographic sampling carried out in October 2017. In the same occasion, the cover of *C*. *amentacea* adults in each experimental plot was also visually estimated with a 20 x 20 cm frame subdivided into 25 sub-quadrats. A score between 0 (absence) and 4 (an entire sub-quadrat covered) was assigned to each sub-quadrat, adding up all the 25 estimates to a final value expressed as percentage [49].

In addition, a destructive sampling was carried out in October 2017 to characterize the benthic assemblage of the study area. Algal assemblages and associated sessile and vagile fauna were collected integrating a pressure vacuum-cleaner connected to a tank and scraping with hammer and chisel. Five 20 x 20 cm randomly placed quadrats were collected at each site. Macroalgal taxa were identified and distinguished into morphological groups, and their abundance was estimated as wet weight (g / 400 cm^2^). In three randomly-chosen samples per site, invertebrates larger than 1 mm were identified at the species level and their abundance was expressed as number of individuals / 400 cm^2^. A literature research allowed to identify the functional traits of the identified species with a focus on their trophic group (S1 Table). In May, July and October 2017 the number of sea urchins (*Paracentrotus lividus* (Lamarck, 1816); and *Arbacia lixula* (Linnaeus, 1758)) was estimated in 20 randomly placed 50 x 50 cm quadrats at each site.

### Statistical analyses

Linear mixed-effects models (LME) were used to evaluate the abundance of germlings surviving during the three days of outplanting in the field, as the method can handle unbalanced data with a hierarchical temporal structure [50]. The number of germlings per tile was transformed in a logarithmic scale to normalize the data [50]. The model included the fixed factors Day and Halfday (nested within Day, indicating morning and afternoon) and Halfday|Day as random effects. The structure of the random term was selected comparing models with different error structures using the Akaike information criterion (AIC, [51]) (S4 Table). Significance of the fixed factors was assessed by means of Wald test [52, 53] (S5 Table). To partition and compare variability among factors, variance components of the number of germlings per tile were estimated among days of outplanting, between halfdays within each day, and among tiles within each halfday with a model including 1 | Day/Halfday as random effects [50]. Model assumptions were assessed by visually inspecting plots of residuals vs. fitted values (S2 Fig). The models were fitted using the function lmer in the R package lme4 [54].

Due to unexpected mortality during the outplanting phase, effects of post-settlement processes (i.e. macrograzing and presence of *C*. *amentacea* adults) were evaluated in two locations (Marittima and Torre Guaceto, respectively one donor and one restoration site). To avoid confounding the influence of manipulated factors (i.e., presence of adult conspecifics and herbivores access) with stochastic effects naturally affecting small populations, we *a-priori* decided to retain only quadrats containing a number of germlings at least equal to 100 at the time of outplanting, ideally guarantying the survival of at least 10 *C*. *amentacea* individuals per quadrat (estimates were based on previous experience gained during a small-scale pilot study). At Marittima and Torre Guaceto, nearly all quadrats satisfied this condition (respectively 34 and 32 units out of 36), while at Sant’Isidoro and Porto Cesareo it was rarely fulfilled (respectively 21 and 3 units out of 36, not homogeneously distributed across experimental conditions). Hence, only Marittima and Torre Guaceto were retained for the analysis of post-settlement processes (i.e. macrograzing and presence of *C*. *amentacea* adults).

Effects of post-settlement processes on the survival of juveniles after three months were assessed with the analysis of variance (ANOVA) on the total number of germlings per quadrat (i.e. summing the values of the 5 tiles), as sampling units were balanced and all assumptions of the analysis were met. The experimental design included four factors (Fig 1): Location (fixed, with two levels, “Marittima” and “Torre Guaceto”), Site (random, with two levels, nested within Location), Adult transplant (fixed, orthogonal to all other factors, with two levels, “Transplanted adults” and “No adults transplanted”) and Herbivory (fixed, orthogonal to all other factors, with three levels, "Herbivores", "No herbivores", "Artifact control"), with n = 3 replicates for each combination of factors. To account for the variability among quadrats in the number of germlings deployed during outplanting, the analysis was repeated including the initial number of germlings as a covariate. However, as the covariate was not significant, and it did not increase the predictive power of the model, results are not shown.

The efficacy of adult transplant was assessed analyzing *C*. *amentacea* cover with ANOVA, hypothesizing that the considered variable would not differ among Conditions (i.e. Donor populations, with unmanipulated *C*. *amentacea*, and Restored populations, where the macroalgae had been transplanted). The experimental design included four factors: Condition (fixed, with two levels, "Donor", representing the unmanipulated control, and "Restoration", including transplanted quadrats), Location (random, with two levels, nested within Condition), Site (random, with two levels, nested within Location), and Herbivory (fixed, orthogonal to all other factors, with three levels "Herbivores", "No herbivores", "Control of artifact"), with n = 3 replicates for each combination of factors. Also, the absence of artifact associated to the transplant technique was verified from the lack of differences between control of artifact treatments (i.e. cross transplant between donor locations, translocation between sites within location and dislocation within site) and unmanipulated controls.

ANOVA was also employed to test patterns of abundance of mesograzers. Species were classified according to their trophic group and the total number of grazers and omnivores belonging to each of the most abundant taxonomical groups (mollusks, amphipods, polychaetes) was analyzed by means of two-way ANOVA. As there was no a-priori hypothesis on differences in patterns of distribution of mesograzers among Conditions, the model was simplified to include Location (random, with 4 levels) and Site (random, with 2 levels nested in Location).

In all ANOVAs, homogeneity of variances was checked with Cochran’s C-test and, when necessary, data were log or square-root transformed (respectively abundance of *Cystoseira* germlings and amphipods). A posteriori Student-Newman-Keuls (SNK) tests were used to compare 1- differences in the abundance of settled *Cystoseira* germlings among levels of Herbivory within each Site, 2- differences in the abundance of mesograzers among Locations, 3- differences in the cover of *Cystoseira* adults among Sites. Analyses of variance were performed in R v3.5 (R Core Team 2013) using the package GAD [55].

To characterize patterns of distribution of macroalgae among study sites, the assemblage structure was analyzed by means of a permutational multivariate analysis of variance (PERMANOVA) [56, 57]. The model included two factors: Location (random, with two levels) and Site (random, with 2 levels nested in Location). Although locations were originally chosen randomly and so treated as a random factor in the analysis, it was nevertheless of interest to assess differences among Locations by using pairwise *a posteriori* comparison, to better interpret patterns of abundance of *Cystoseira* germlings after three months. Permutational analysis of multivariate dispersion (PERMDISP) was performed to evaluate the homogeneity of multivariate dispersion among locations. Analyses were done on Bray-Curtis dissimilarity [57, 58] matrix of non-transformed wet weight of macroalgal species in five 20x20 cm quadrats for each site. Similarity percentage analysis (SIMPER) identified the percentage contribution of each macroalgal species to the Bray-Curtis dissimilarity metric between pairs of locations. A 2-dimensional MDS (multidimensional scaling) based on Bray-Curtis measures of dissimilarity was used for a graphical representation of the multivariate structure of macroalgal assemblage. Multivariate analyses have been carried out using PRIMER version 6, including the add-on package PERMANOVA+ [53].

### Cost analysis

The cost of restoration intervention was calculated separately for the nursery phase in the laboratory and for the set-up and outplanting in the field. Similar to the approach by Carney et al. [59] and Verdura et al. [60], transportation of fertile apices and recruits, travel, equipment and personnel expenses were evaluated. Also, an evaluation of the monitoring cost for one date of sampling was provided. Travel expenses were estimated equal to 0.21 €/km or to 0.27 €/km when highway toll was included, while the personnel cost was estimated equal to 7 € hour/person.

## Results

### Effect of experimental conditions on germlings

**Fig 2:**
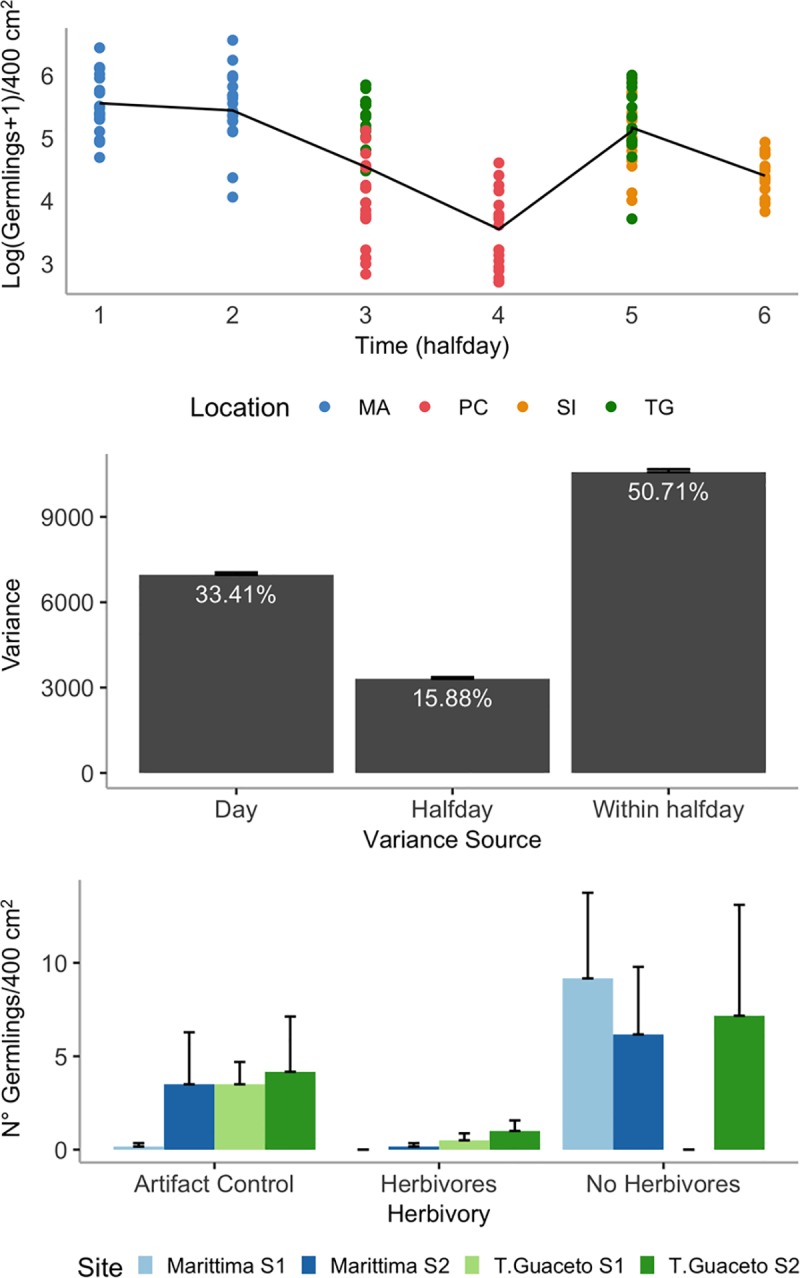
Mortality of germlings during outplanting and in the early stages of growth. (A) Log-transformed number of germlings per tile outplanted in different halfdays of the outplanting phase. Tiles outplanted in different locations are shown with dots of different color. The black line represented the predicted linear mixed-effect model. (B) Variance components of the number of germlings per tile during the outplanting phase. Variance is estimated among days of outplanting, between halfdays within each day and among tiles within each halfday. (C) Number of germlings per quadrat in different Herbivory conditions at different sites after 3 months from outplanting.

At the end of the nursery phase, tiles were colonized by an average number of 450.65 germlings (SE ± 47.04, n = 194). During transports, the average number of germlings per tile reduced to 34.25 (SE ± 1.45, n = 720). The number of germlings surviving during the outplanting process decreased proportionally to the day of placement in the field and in the afternoon compared to the morning within each day (Fig 2A, S5 Table). Although nearly half of the variability in the number of germlings among tiles (50.71%) was due to factors other than the time of outplanting, the partitioning of variance in Fig 2B indicates that it was partially explained by the time elapsed before outplanting (variability among days 33.41%; within the same day 15.88%).

### Effect of *Cystoseira* adults and herbivory on germlings

Three months after settlement in the field, the mean number of germlings per tile had decreased to 0.69 (SE ± 0.12, n = 307), and 53 out of 360 tiles had been lost. Grazers exclusion differently affected the abundance of *C*. *amentacea* germlings among experimental sites (*Herbivory* X *Site* (*Location*) MS = 1.678, F_4,48_ = 2.718, P < 0.05; Fig 2C, S6 Table). At Marittima, where the overall abundance of germlings was greater (*Location* MS = 6.357, F_1,2_ = 39.153, P < 0.05), germlings were favored by grazer exclusion, while at Torre Guaceto a similar trend, although not significant at the SNK test, was clearly observed only at site 2 (Fig 2C, S4 Table). Abundance of germlings was not influenced by the presence of adult conspecifics (Adult Transplant MS = 5.052, F_1,2_ = 5.657, P = 0.140; S6 Table), although transplant of *C*. *amentacea* adults was efficient at maintaining canopy cover comparable between donor and restoration sites (S1 Appendix, S3 Fig and S7 Table).

**Fig 3:**
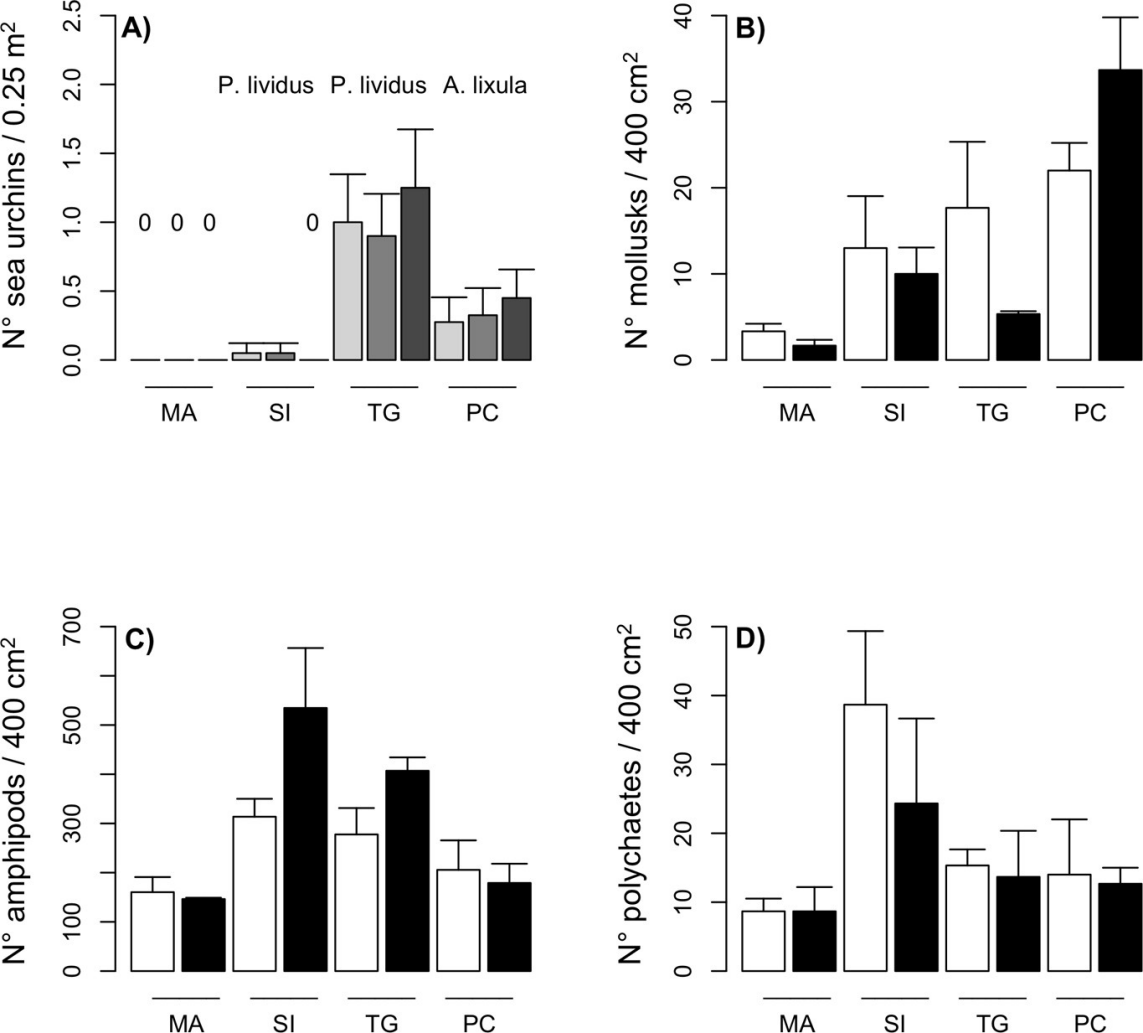
Abundance of macro and mesograzers at experimental sites. (A) Mean number of sea urchins in 50 x 50 cm quadrats in different locations and at different times during the experiment. Light grey = May 2017; middle grey = July 2017, dark grey = October 2017, N = 240. Mean number of (B) mollusks, (C) amphipods and (D) polychaetes in 20 x 20 cm quadrats in different locations and at different sites. MA = Marittima, SI = Sant’Isidoro, TG = Torre Guaceto, PC = Porto Cesareo, black and white bars represent the two sites for each location, N = 24.

Multivariate analyses evidenced differences among locations in the structure of macroalgal assemblage (S2 Table, S1 Fig). Beyond *C*. *amentacea*, the majority of the macroalgae mostly contributing to differentiate assemblages among locations were species mechanically resistant to grazing (i.e. *Ellisolandia elongata*, *Jania rubens*, *Corallina officinalis*, *Titanoderma pustulatum*, *Halimeda tuna*, S3 Table). Also, the analyses on the patterns of distribution of macro and mesograzers evidenced differences among locations. Sea urchins, either *P*. *lividus* or *A*. *lixula*, were constantly present only in the locations where *C*. *amentacea* had naturally disappeared (i.e. restoration sites, Fig 3A). Significant differences among locations were detected also for mesograzers in such taxa as mollusks (*Location* MS = 668.7, F_3,4_ = 5.94, P < 0.05, Fig 3B) and polychaetes (*Location* MS = 598.8, F_3,4_ = 7.60, P < 0.05, Fig 3D), but not for amphipods (*Location* MS = 84.9, F_3,4_ = 5.52, P = 0.06; Fig 3C). SNK tests for mollusks and polychaetes evidenced a lower number of grazers at Marittima compared to other experimental locations, and a similar trend, although without significant differences, was evident also for amphipods. The most abundant herbivores among mollusks were the chitons *Callochiton septemvalvis* (Montagu, 1803), *Lepidochitona monterosatoi* Kaas & Van Belle, 1981, and *Acanthochitona fascicularis* (Linné, 1767), while polychaetes were dominated by *Platynereis dumerilii* Audouin & Milne-Edwards, 1834 and amphipods by *Protohyale schmidtii* (Heller), 1866 and *Ampithoe ramondi* Audouin, 1826.

### Cost analysis

The total cost of the tested restoration approach and follow-up activity was estimated equal to 19755 € (Table 1). The total number of working hours corresponded to 240 in the laboratory and 1598 in the field (including 48 hours for the collection and processing of fertile apices, 170 hours for cages construction, 876 hours for cages fixing, 292 hours for the transplant of *Cystoseira* adults and 212 hours for the installation of germlings tiles). Beside the personnel cost, which corresponded to 66%, the equipment consistently represented the highest source of costs, corresponding to the 22% of the total for the intervention. The activities related to herbivory limitation (cages construction and installation) and adult transplant respectively contributed for 45% and 16% of the total cost.

**Table 1:** Total costs of tested restoration intervention. Pax = person.

Activity		Rate	Cost	Total
**Fertile apices transfer to nursery laboratory**				
Equipment				36.5 €
Personnel		6 h/ 8 pax	7 €/ h*pax	336.0 €
Boat		1 day	170 €/ day	170.0 €
Travel		124 km	0.21 €/ km	26.0 €
Mail delivery				187.0 €
**Laboratory**				
Equipment				1800.0 €
Personnel		5 h/ 24 days * 2 pax	7 €/ h*pax	1680.0 €
**Germlings transfer from nursery to restoration region**				
Personnel		12 h/ 2 pax	7 €/ h*pax	168.0 €
Travel		1115 km	0.27 €/ km	234.2 €
**Field set up**				
Equipment				
	Hardware	96 cages	10.70 €/ item	1031.0 €
	Epoxy	20 kg	55 €/ kg	1103.0 €
	Diving drill	1 item	500 €	500.0 €
Personnel				
	Cages construction	85 h/ 2 pax	7 €/ h*pax	1190.0 €
	Cages installation	219 h/ 4 pax	7 €/ h*pax	6146.0 €
	Adult transplant	73 h/ 4 pax	7 €/ h*pax	2044.0 €
	Germlings outplanting	53 h/ 4 pax	7 €/ h*pax	1512.0 €
Travel		3335 km	0.21 €/ km	700.4 €
**Monitoring**				
Personnel		20 h/ 2 pax	7 €/ h*pax	280.0 €
Travel		354 km	0.21 €/ km	74.4 €
Photo analysis		80 h/ 1 pax	7 €/ h*pax	560.0 €
**Total**				19778.8 €

## Discussion

The present study is the first attempt aimed to restore *C*. *amentacea* beds over a scale of tens of kms, through extensive outplanting of germlings cultured in aquaria. While this active restoration approach offers several advantages compared to transplantation of adults (e.g., availability of large amounts of individuals with negligible impact for extant macroalgal beds), there is the urgency to identify the procedural steps potentially limiting a tangible scale up of intervention. We separately evaluated how germlings survival was affected by experimental conditions, presence of adult conspecifics and herbivory, including the consideration of the associated costs. The study was featured by a limited number of sites, driven by the challenge of a very intense field work, and the total duration was limited to three months, which is a short time to fully evaluate the success of the restoration actions. However, our intention was to quantitatively describe those steps in the early stages of restoration interventions making restoration particularly vulnerable, as a mandatory knowledge to set future large-scale interventions.

### Effect of experimental conditions on germlings mortality

Mortality of germlings during the restoration intervention was very high in each phase: only 7% of individuals grown in the nursery reached restoration locations and only 2% of them survived the initial settlement stage. In the literature, high mortality rates are normally encountered by macroalgal recruits [61, 62], and low survival rates in the first year have been reported also for *Cystoseira* sp. [63, 64]. Schiel et al. [41] estimated survival rates from embryos to visible recruits variable from 0.001% to 3.8%. Besides, the first 2–3 months since recruits become visible represent a critical phase, accounting for nearly 40–50% of the mortality occurring within 3 years [40, 41].

Germlings mortality can be density-dependent [40, 41], and intraspecific competition might be exacerbated by limited nutrient availability and elevated temperatures [65]. High temperature itself may reduce survival of juvenile macroalgae [66–68], although undesirable effects have not been detected for the growth of *Cystoseira barbata* juveniles [42]. Yet, the temperature range experimentally investigated by Irving et al. [42] simulates naturally occurring spring values (16°), which might result less stressing compared to the extreme temperature values tested by other authors [65–68], better resembling the stressful summer conditions occurring in our restoration experiment. Although germlings were constantly refrigerated during transportation and outplanting, temperature stress might justify the high mortality and in particular the increase in mortality observed for individuals placed in the field in the afternoon compared to those outplanted in the morning, since solar heat can represent a stress during attachment.

Our study evidences increased mortality for germlings stored in the laboratory for prolonged time (24–48 hours before outplanting) and a large percentage of variability in the number of germlings per tile. Experimental conditions maintained in the laboratory are crucial to reduce mortality, representing also a cost as shown by Verdura et al. [60]. Also, abrupt variations of environmental conditions and mechanical damage during transportation or manipulation might have contributed to the mortality of germlings before and during the settlement in the field. Our results show that the availability of nursery facilities in close proximity to restoration sites can significantly contribute to the success of intervention. This is a critical point considering present and future need of restoration actions in areas of the world where facilities are still lacking. In particular, the identification of strategies to minimize mortality during transport and outplanting represents a priority for future interventions. Specific experiments evaluating how vulnerability of recruits to abiotic stress varies with age could indicate the optimal duration of laboratory cultivation phase and the eventual advantage due to postponing the outplanting in the field.

### Effect of *Cystoseira* adults on germlings mortality

Survival of *Cystoseira* germlings settled in the field was not influenced by the presence of adult conspecifics. Positive and neutral intraspecific interactions have been found common in seaweed communities globally [69]. In particular, recruitment occurring preferentially below canopy fronds or at the edge of canopy beds has been documented for several forest-forming macroalgae, both in the intertidal and in subtidal habitats [23, 70]. The presence of adult conspecifics creates favorable conditions for the settlement of juveniles, by protecting recruits form grazers or competitors [71, 72] or by ameliorating abiotic environmental conditions [73, 74]. In particular, in the intertidal fringe, desiccation represents a major source of stress for macroalgae, as it can impair photosynthetic activity, reduce growth or increase brittleness and mortality due to dislodgement by waves [75]. However, desiccation stress could have exerted a minor role at our study sites, where *C*. *amentacea* colonized vermetid-platforms or rocky coastal belts at depths virtually constantly submerged by water. In contrast, the presence of canopy fronds is known to inhibit recruitment of several kelp and fucoids species ([40] and references therein; [76]), and neutral or negative effects have been recorded for different *Cystoseira* species inhabiting subtidal reefs [18, 28, 43, 63]. Algal canopy may negatively affect the survival of germlings through frond abrasion [40] or by reducing light irradiance up to 95–99%, a resource of primary importance for the development and growth of *Cystoseira* recruits [42]. Variation in the outcomes of intraspecific interactions is clearly common in nature and can change as a function of the biotic and/or abiotic context.

### Effect of herbivory on germlings mortality

Preventing the access of macrograzers by caging successfully guaranteed the survival of germlings in our study, while the absence of protection led to nearly null survival (0.42 ± 0.17 individuals per quadrat). Overgrazing is known to be an important cause of decline of macroalgal forests worldwide and represents the primary cause for unsuccess in macroalgal restoration intervention, with drastic effects described for kelp-forming macroalgae [59, 72] and *Cystoseira* species [20, 35]. Signs of grazing by salema fish have been observed on transplanted adult individuals of *C*. *amentacea* and *C*. *compressa* by Susini et al. [26]. Mangialajo et al. [27] suggested that salema fish may consume also *C*. *amentacea* recruits. Salema fish is a very voracious herbivore, capable to graze also on intertidal reefs, to reduce biomass up to 90%, and to decrease fertility by preferentially consuming apices with receptacles [34, 37]. At our study sites, salema schools were frequently observed grazing on the vermetid platform, and signs of bites were noticed on uncaged transplanted adults. Control of artifact cages had windows to allow entrance by grazers, although the complexity of the structure might have prevented grazing by salema fish. Hence, we can suppose that the amount of germlings survived into control of artifacts might represent the number of individuals that survived salema grazing and was impacted only by other herbivores.

Similar to our study, experiments aiming at introducing *Cystoseira* spp. on artificial reefs successfully adopted caging to prevent grazing of juveniles or displacement and damaging due to non-consumptive biotic interactions (i.e. handling and clipping of thalli; [28, 77]). These experiments evidenced that, in addition to the impact of salema fish, juvenile survival might be threatened by several herbivores and omnivores species, such as mullets, crustaceans (e.g. crabs) or mollusks (e.g. limpets) [34, 77]. The sea urchins *P*. *lividus* and *A*. *lixula*, although scarcely abundant in intertidal rocky shores, may severely reduce the survival and prevent the recovery of *Cystoseira* in proximity of sea urchins’ refugees [78]. At our study sites, sea urchins likely exerted a prominent role in determining patterns of distribution of *C*. *amentacea*, as they were present only in locations were *C*. *amentacea* had historically disappeared. Densities of *A*. *lixula* in Porto Cesareo were nearly half compared to those of *P*. *lividus* in Torre Guaceto. However, *A*. *lixula* is very efficient at maintaining barren habitats, and even low densities can prevent *Cystoseira* spp. recovery [31, 79].

Other species might be efficient grazers of the small-size, fragile *Cystoseira* germlings. A high pressure of herbivores at restoration sites (i.e. Porto Cesareo and Torre Guaceto) and in Sant’Isidoro is suggested by the abundance of articulated corallinales (e.g. *Jania rubens* and *Ellisolandia elongata*) or of other algal species (e.g. *Halimeda tuna*) which are mechanically resistant to grazing and scarcely appealing for herbivores [80]. Analyses of the abundance of mesograzers confirmed a reduced presence of herbivore mollusks and polychaetes at Marittima, where we observed the greater survival of *Cystoseira* germlings. Also, the highest abundance of mollusks at one site in Torre Guaceto corresponded to the lowest survival of *Cystoseira* germlings, suggesting grazing as a plausible cause of mortality. Effects of mesograzers on macroalgal forests have rarely been studied [81], possibly due to the difficulties in manipulating them in the field for experimental purposes. However, some studies have shown that small-size species, such as amphipods or gastropods, may be crucial in regulating the success of recruitment of Laminariales or Fucales [82–84]. In some species of macroalgae, juveniles, having lower content of polyphenolic compounds, may be more palatable for mesograzers than adult conspecifics [85]. *Ad hoc* experimental studies in the laboratory would help elucidating the overlooked impact of mesograzers on early post-settlement stages of *Cystoseira*, although the small size and high mobility of the herbivorous do not allow to implement strategies for reducing their abundance in the field for restoration purposes. While a characterization of the fish and the benthic assemblages at putative restoration sites may contribute to discard unappropriated locations, the impact of mesograzers should be reckoned as a unavoidable, further cause of mortality for juveniles.

### Cost analysis

As demonstrated, 135 germlings survived in the optimal conditions (locations with timely outplanting after transport from the nursery and macrograzer exclosure, independently from the presence of adult conspecifics). If resources would have been allocated for all 144 experimental units to this condition, we could have grown nearly 810 germlings, with an estimated cost per individual ~ 23.30 €. This value is nearly double the cost estimated for the culturing and outplanting of *Nereocystis luetkeana* by Carney et al. [59]. Yet, in their study grazing contributed to the failure of juvenile transplant intervention, and the authors advocated for the use of grazer exclusion devices in sites were grazers are abundant, with consequent increase of the cost of intervention per individual. In our intervention, limitation of herbivory severely conditioned the duration and cost of intervention, requiring 55% of working hours in the field and 45% of total expenses. Recently, Verdura et al. [60] compared the cost of restoring a population of *Cystoseira barbata* using the *ex situ* and *in situ* methods, considering the travel, transportation, personnel and material expenses. Similar to Carney et al. [59], they did not use any structure to limit herbivory, as the site did not require them. They concluded that the cost of restoring 25 m^2^ of this species corresponded to 2665 € using the *ex situ* seeding technique, with an estimated cost per individual ~ 25.60 €, for a total of ~ 104 plants per site. Although the cost is comparable to our intervention, it is worth noting that their cost-benefit analysis is based on the success of the method in a long-term assessment over 6 years, reaching an adult, self-sustaining population.

## Conclusions

**Fig 4:**
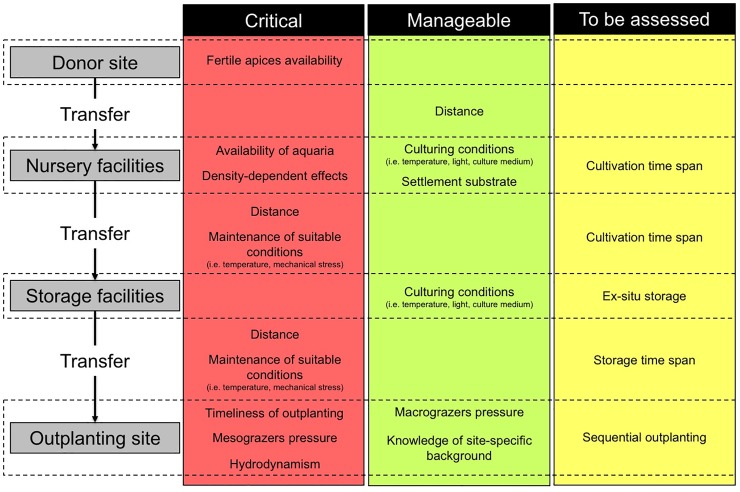
Schematic representation of *C*. *amentacea ex-situ* cultivation and outplanting. For each step of the restoration intervention, the red box synthetizes issues limiting its feasibility or representing sources of mortality for *C*. *amentacea* germlings, according to the present study. The green box presents manageable issues, according to the strategies adopted in the present study and described in the literature. In the yellow box, issues that still require to be addressed are introduced, as promising strategies to overcome criticalities.

Our attempt to operate over large spatial scales required the cultivation of a high number of individuals (approximately 325,000 at the end of the nursery phase), and raised several methodological issues during the outplanting process. Processes deserving attention and still requiring further research when approaching *Cystoseira* restoration are summarized in Fig 4. Availability of fertile receptacles influences the timing of both *ex situ* and *in situ* restoration, as the suitable season might be limited to 2–3 months per year and varies according to the species, to climatic conditions, and to the geographical position of the donor location. The availability of appropriate facilities limits the number of individuals grown, and excessive densities of germlings in the aquaria may result in negative density-dependent effects such as competition or blooms of epiphytes or pathogenic agents (e.g. bacteria). Species-specific culturing conditions, optimal duration of the cultivation phase, and the appropriate substratum need to be identified with proper experimentation, similarly to the approach described by Falace et al. [33].

One of the most delicate phases is transport from the nursery and storage location to the field, as juveniles are susceptible to variations in abiotic conditions and to mechanical stress. Also, timeliness in the outplanting process is crucial and guidelines for the handling of germlings in the delicate gap between the nursery and the field could be further implemented with new strategies. For example, the temporal desynchronization of germlings cultivation could provide a manageable number of germlings to be outplanted in several subsequent occasions, possibly minimizing germlings mortality. Alternatively, areas for the storage and acclimation of germlings prior to attachment in the field could be individuated in enclosed coastal areas, eventually allowing for a longer time of cultivation in semi-controlled conditions, in order to minimize size-dependent mortality due to stressful conditions (e.g. abiotic and mesograzing pressures). Once in the field, the presence of adult conspecifics does not represent a necessary pre-requisite for the survival of *C*. *amentacea* germlings. In contrast, the mitigation of grazing pressure is crucial and could be implemented by setting up protective cages, although these structures cannot prevent the access to mesograzers and their application could be critical over vast extensions (high costs, high probability of dislodgement by waves, use of plastic, esthetic injury). Pilot studies carried out on putative restoration sites should be considered a priority to individuate promising locations according to abiotic (e.g. hydrodynamic conditions) and biotic criteria (e.g. frequentation by macrograzers, structure of mesograzers assemblage). Yet, climate change is expected to severely affect the recruitment and survival of *Cystoseira* germlings, either directly or indirectly (e.g. increasing grazing rates), eventually setting new priorities for the success of restoration interventions. For example, the negligible presence of *Cystoseira* adults might become a favourable, necessary condition under warming climate [69]. Also, macrograzers pressure is expected to increase [86], although no specific predictions have been formulated for the activity of mesograzers.

Taken together, our analyses show that large-scale restoration is possible only if basic information about species, protocols and the areas to be restored is carefully addressed. At the moment, specific details about ecophysiology are available only for a few *Cystoseira* species (*C*. *amentacea* var. *stricta*, [26, 27, 33]; *C*. *barbata*, [9, 18, 25, 28, 29, 42, 60, 77]; *C*. *compressa*, [25–27, 77]; *C*. *zosteroides*, [63, 64, 68, 87]) out of 45 *Cystoseira* species existing in the Mediterranean Sea [88], limiting the potential of selecting suitable species in a restoration framework. Although the Mediterranean Action Plan, adopted within the framework of the Barcelona Convention (1976), identifies, in an amendment of 2009 (Annex IV, SPA/BD Protocol—United Nations Environment Programme [UNEP]), the conservation of all but one Mediterranean *Cystoseira* species (*C*. *compressa*) as a priority, most *Cystoseira* forests are under severe threat.

Restoring areas depleted by macroalgal forests is an important target given the shifts that we are documenting at global scales. However, at this stage, a priority should be to address efforts and resources devoted to this activity would be better spent in mitigating local stressors and preserving existing forests. A better coordination among complementary strategies of mitigation, conservation and restoration seems to be still the best strategy to halt present trajectories of changes.

## Supporting information

S1 Appendix Efficacy of *C. amentacea* adult transplant: statistical analyses. (DOCX)

S1 Table Structure of benthic assemblage at experimental sites: invertebrates trophic group. List of Mollusca, Amphipoda and Polychaeta species sampled at experimental sites. Information on their trophic group were collected from the literature.
(DOCX)

S2 Table Structure of macroalgal assemblage at experimental sites: PERMANOVA and PERMDISP. Permutational analysis of variance (PERMANOVA) on the structure of macroalgal assemblages in different conditions (Donor and Restoration locations), at different locations (nested within conditions) and sites (nested within locations). PERMDISP analysis and pairwise comparisons were used to evaluate the homogeneity of multivariate dispersion among locations. Analyses were done on Bray-Curtis dissimilarity matrix of non-transformed wet weight of macroalgal species in 5 quadrats for each site. SI = Sant’ Isidoro (Donor); MA = Marittima (Donor); TG = Torre Guaceto (Restoration); PC = Porto Cesareo (Restoration). ** P < 0.01.
(DOCX)

S3 Table Structure of macroalgal assemblage at experimental sites: SIMPER. Similarity percentage analysis (SIMPER) identifying the % contribution of each macroalgal species (g wet weight scraped from 400 cm^2^ quadrats) to the Bray Curtis dissimilarity metric between pairs of locations. Average dissimilarity: Sant’ Isidoro (SI)—Marittima (MA) = 57.04; Sant’ Isidoro—Torre Guaceto (TG) = 81.64; Marittima—Torre Guaceto = 84.22; Sant’ Isidoro—Porto Cesareo (PC) = 74.19; Marittima—Porto Cesareo = 79.82; Torre Guaceto—Porto Cesareo = 64.86.
(DOCX)

S4 Table Comparison among linear mixed-effects models assessing the survival of germlings during outplanting. The structure of the random term was selected comparing models with different error structures using the Akaike information criterion (AIC).
(DOCX)

S5 Table Factors influencing survival of *C. amentacea* germlings during outplanting. Linear mixed-effects model assessing the survival of germlings during outplanting. The number of germlings per tile was log-transformed to normalize the data. Significance of the fixed factors was assessed by mean of the Wald test.
(DOCX)

S6 Table Factors influencing survival of *C. amentacea* germlings during early settlement phases. Analysis of variance of the effects of *C*. *amentacea* adults and herbivory due to macrograzers on the number of germlings per quadrat survived at different sites and locations after three months. The sum of germlings in the five tiles within each quadrat was log-transformed. Cochran’s C = 0.082. He = free access from macrograzers, No He = cages of grazers exclosure, CA = control of artifact. * *P* < 0.05.
(DOCX)

S7 Table Efficacy of *C. amentacea* adult transplant: ANOVA. Analysis of variance on the abundance of *C*. *amentacea* adults (cover / 400 cm^2^ quadrats) among different herbivory treatments (free access from macrograzers, grazers exclosure, control of artifact) in different conditions (transplanted at restoration sites and unmanipulated at donor sites), at different locations (two levels, nested within condition) and sites (two levels, nested within location). Cochran’s C = 0.185. = 0.191 ** *P* < 0.01.
(DOCX)

S1 Fig Structure of macroalgal assemblage at experimental sites: MDS. Multidimensional scaling (MDS) of the structure of macroalgal assemblage at experimental locations. SI = Sant’Isidoro (Donor); MA = Marittima (Donor); TG = Torre Guaceto (Restoration); PC = Porto Cesareo (Restoration).
(TIFF)

S2 Fig Plot of residuals of selected linear mixed model. Plot of the residuals showing the linearity and normality of the data after the logarithmic transformation of the number of germlings per tile.
(TIF)

S3 Fig Efficacy of *C. amentacea* adult transplant in October 2017. Cover of *C*. *amentacea* adults at experimental sites in October 2017. Light grey = site 1; dark grey = site 2 within each location, N = 144.
(TIFF)
