# Supplementary material for: Are we ready for scaling up restoration actions? An insight from Mediterranean macroalgal canopies
Source: PLoS One. 2019 Oct 25;14(10):e0224477. doi: 10.1371/journal.pone.0224477 (PMC6814225; doi:10.1371/journal.pone.0224477)
Supplement: S1 Appendix — (DOCX) [file pone.0224477.s001.docx]

**S1 Appendix. Efficacy of *C. amentacea* adult transplant: statistical analyses.**

Analysis of variance was run including only quadrats were *C. amentacea* adults were naturally present (donor locations) or were transplanted (restoration locations), to evaluate the efficacy of adult transplant. Herbivory treatment was included in the analysis to evaluate eventual artifacts due to the present of cages on the persistence of transplanted individuals.

At that time of the year, only bases of thalli are present, as fronds are removed by hydrodynamic forces by the end of the summer.
