# Supplementary material for: Are we ready for scaling up restoration actions? An insight from Mediterranean macroalgal canopies
Source: PLoS One. 2019 Oct 25;14(10):e0224477. doi: 10.1371/journal.pone.0224477 (PMC6814225; doi:10.1371/journal.pone.0224477)
Supplement: S1 Table — List of Mollusca, Amphipoda and Polychaeta species sampled at experimental sites. Information on their trophic group were collected from the literature. (DOCX) [file pone.0224477.s002.docx]

**S1 Table**. **Invertebrates trophic group**

List of Mollusca, Amphipoda and Polychaeta species sampled at experimental sites. Information on their trophic group were collected from the literature.

| **Polychaeta** | **Trophic group** |
| --- | --- |
| *Platynereis dumerilii* (Audouin & Milne-Edwards, 1834) | Herbivorous |
| *Perinereis cultrifera* (Grube, 1840) | Omnivorous |
| *Neanthes fucata* (Savigny, 1822) | Omnivorous |
| *Platynereis nadiae* Abbiati & Castelli, 1992 | Unknown - Mobile |
| *Perinereis oliveirae* (Horst, 1889) | Unknown - Mobile |
| *Amphiglena mediterranea* (Leydig, 1851) | Filter-feeder |
| *Eteone picta* (Quatrefages, 1866) | Predator - Scavenger |
| *Sabellaria alveolata* (Linnaeus, 1767) | Filter-feeder |
| *Serpula verticillata* Spalowsky, 1795 | Filter-feeder |
| *Lumbrineris sp.* | Predator |
| *Caulleriella viridis* (Langerhans, 1881) | Unknown - Mobile |
| *Caulleriella bioculata* (Keferstein, 1862) | Unknown - Mobile |
| *Syllis spp.* | Omnivorous |
| *Amphicorina sp.* | Filter-feeder |
| *Vermiliopsis striaticeps* (Grube, 1862) | Filter-feeder |
| *Vermiliopsis labiata* (O. G. Costa, 1861) | Filter-feeder |
| *Lysidice ninetta* Audouin & Milne-Edwards, 1833 | Unknown - Mobile |
| *Lysidice unicornis* (Grube, 1840) | Unknown - Mobile |
| *Lysidice collaris* Grube, 1840 | Unknown - Mobile |
| *Euniphysa italica* Cantone & Gravina, 1991 | Unknown - Mobile |
| *Ceratonereis hircinicola* (Eisig, 1870) | Unknown - Mobile |
| **Mollusca** |  |
| *Ischnochiton (Ischnochiton) rissoi* (Payraudeau, 1826) | Herbivorous |
| *Callochiton septemvalvis* (Montagu, 1803) | Detritus feeder - Herbivorous |
| *Lepidochitona sp* | Herbivorous |
| *Lepidochitona monterosatoi* Kaas & Van Belle, 1981 | Herbivorous |
| *Chiton (Rhyssoplax) olivaceus* (Spengler, 1797) | Herbivorous |
| *Acanthochitona crinita crinita* (Pennant, 1777) | Omnivorous |
| *Acanthochitona fascicularis* (Linné, 1767) | Omnivorous |
| *Patella ulyssiponensis* Gmelin, 1791 | Herbivorous |
| *Diodora gibberula* (Lamarck, 1822) | Herbivorous |
| *Diodora gibberula* (Lamarck, 1822) | Herbivorous |
| *Sinezona cingulata* (Costa O.G., 1861) | Herbivorous |
| *Tricolia pullus pullus* (Linné, 1758) | Herbivorous |
| *Tricolia tenuis* (Michaud,1829) | Herbivorous |
| *Clanculus (Clanculopsis) cruciatus* (Linné, 1758) | Detritus feeder |
| *Gibbula (Gibbula) ardens* (Von Salis, 1793) | Detritus feeder |
| *Gibbula (Colliculus) turbinoides* (Deshayes, 1835) | Detritus feeder |
| *Gibbula (Steromphala) divaricata* (Linné, 1758) | Herbivorous |
| *Gibbula (Tumulus) umbilicaris umbilicaris* (Linné, 1758) | Herbivorous |
| *Phorcus turbinatus* (Von Born, 1778) | Detritus feeder - Herbivorous |
| *Eatonina (Coriandria) cossurae* (Calcara, 1841) | Detritus feeder |
| *Eatonina (Coriandria) fulgida* (Adams J., 1797) | Detritus feeder |
| *Rissoa similis* Scacchi, 1836 | Herbivorous |
| *Rissoa variabilis* (Von Muehlfeldt, 1824) | Herbivorous |
| *Alvania (Alvania) cancellata* (Da Costa, 1778) | Herbivorous |
| *Alvania (Alvania) mamillata* Risso, 1826 | Detritus feeder - Herbivorous |
| *Alvania (Alvania) pagodula* (Bucquoy, Dautzenberg & Dollfus, 1884) | Detritus feeder - Herbivorous |
| *Alvania (Galeodina) tenera* (Philippi, 1844) | Detritus feeder - Herbivorous |
| *Crisilla semistriata* (Montagu, 1808) | Detritus feeder - Herbivorous |
| *Pusillina philippi* (Aradas & Maggiore, 1844) | Detritus feeder - Herbivorous |
| *Setia amabilis* (Locard, 1886) | Detritus feeder - Herbivorous |
| *Setia turriculata* Monterosato, 1884 | Detritus feeder - Herbivorous |
| *Barleia unifasciata* (Montagu, 1803) | Detritus feeder - Herbivorous |
| *Cerithium scabridum* Philippi, 1848 | Detritus feeder - Herbivorous |
| *Cerithium vulgatum* Bruguière, 1792 | Detritus feeder - Herbivorous |
| *Bittium jardetinum* (Brusina, 1865) | Detritus feeder |
| *Bittium latreilli* (Payraudeau, 1826) | Detritus feeder |
| *Bittium reticulatum* (Da Costa, 1778) | Detritus feeder - Herbivorous |
| *Vermetus (Vermetus) triquetrus* Bivona Ant., 1832 | Filter-feeder |
| *Vermetus (Thylacodus) granulatus* (Gravenhorst, 1831) | Filter-feeder |
| *Dendropoma cristatum* (Biondi, 1857) | Filter-feeder |
| *Melaraphe neritoides* (Linné, 1758) | Herbivorous |
| *Cerithiopsis minima* (Brusina,1865) | Predator |
| *Cerithiopsis tubercularis* (Montagu, 1803) | Predator |
| *Dizoniopsis coppolae* (Aradas, 1870) | Predator |
| *Similiphora similior* (Bouchet & Guillemot, 1978) | Predator |
| *Ocinebrina edwardsii* (Payraudeau, 1826) | Predator |
| *Pisania striata* (Gmelin, 1791) | Predator |
| *Pollia dorbignyi* (Payraudeau, 1826) | Predator |
| *Pollia scacchiana* (Philippi, 1844) | Predator |
| *Columbella rustica* (Linné, 1758) | Herbivorous |
| *Nassarius (Telasco) cuvierii* (Payraudeau, 1826) | Detritus feeder |
| *Gibberula miliaria* (Linné, 1758) | Predator |
| *Vexillum (Pusia) ebenus* (Lamarck, 1811) | Predator |
| *Mangelia taeniata* (Deshayes, 1835) | Predator |
| *Clathrella clathrata* (Philippi, 1844) | Parasite |
| *Chrysallida incerta* (Milaschewitch, 1916) | Predator |
| *Berthella aurantiaca* (Risso, 1818) | Unknown |
| *Arca noae* Linné, 1758 | Filter-feeder |
| *Barbatia (Barbatia) barbata* (Linné, 1758) | Filter-feeder |
| *Mytilus galloprovincialis* Lamarck, 1819 | Filter-feeder |
| *Mytilaster minimus* (Poli, 1795) | Filter-feeder |
| *Modiolarca subpicta* (Cantraine, 1835) | Filter-feeder |
| *Musculus costulatus* (Risso, 1826) | Filter-feeder |
| *Lithophaga lithophaga* (Linné, 1758) | Filter-feeder |
| *Ostrea edulis* Linné, 1758 | Filter-feeder |
| *Spondylus (Spondylus) gaederopus* Linné, 1758 | Filter-feeder |
| *Anomia ephippium* Linné, 1758 | Filter-feeder |
| *Cardita calyculata* (Linné, 1758) | Filter-feeder |
| *Glans (Glans) trapezia* (Linné, 1767) | Filter-feeder |
| *Chama gryphoides* Linné, 1758 | Filter-feeder |
| *Hiatella arctica* (Linné, 1767) | Filter-feeder |
| ***Amphipoda*** |  |
| *Apolochus cfr. neapolitanus* (Della Valle, 1893) | Predator |
| *Tritaeta gibbosa* (Spence Bate, 1862) | Omnivorous |
| *Stenothoe monoculoides* (Montagu, 1815) | Omnivorous |
| *Stenothoe tergestina* (Nebeski, 1881) | Omnivorous |
| *Leucothoe sp.* | Unknown |
| *Caprella cavediniae* Krapp-Schickel & Vader, 1998 | Omnivorous |
| *Caprella hirsuta* Mayer, 1890 | Omnivorous |
| *Caprella liparotensis* Haller, 1879 | Predator - Detritus feeder |
| *Podocerus variegatus* Leach, 1814 | Detritus feeder |
| *Ischyrocerus inexpectatus* Ruffo, 1959 | Detritus feeder |
| *Jassa ocia* (Spence Bate, 1862) | Detritus feeder |
| *Jassa sp.* | Detritus feeder |
| *Ericthonius punctatus* (Spence Bate, 1857) | Detritus feeder |
| *Gammaropsis crenulata* Krapp-Schickel & Myers, 1979 | Detritus feeder |
| *Lembos websteri* Spence Bate, 1857 | Detritus feeder - Omnivorous |
| *Leptocheirus guttatus* (Grube, 1864) | Detritus feeder |
| *Microdeutopus cfr. similis* Myers, 1977 | Detritus feeder |
| *Ampithoe ramondi* Audouin, 1826 | Herbivorous |
| *Ampithoe sp.* | Herbivorous |
| *Elasmopus spp.* | Detritus feeder - Omnivorous |
| *Quadrimaera spp.* | Detritus feeder |
| *Protohyale schmidtii* (Heller, 1866) | Herbivorous |
| *Pereionotus testudo* (Montagu, 1808) | Omnivorous |
