# Supplementary material for: Are we ready for scaling up restoration actions? An insight from Mediterranean macroalgal canopies
Source: PLoS One. 2019 Oct 25;14(10):e0224477. doi: 10.1371/journal.pone.0224477 (PMC6814225; doi:10.1371/journal.pone.0224477)
Supplement: S2 Table — Permutational analysis of variance (PERMANOVA) on the structure of macroalgal assemblages in different conditions (Donor and Restoration locations), at different locations (nested within conditions) and sites (nested within locations). PERMDISP analysis and pairwise comparisons were used to evaluate the homogeneity of multivariate dispersion among locations. Analyses were done on Bray-Curtis dissimilarity matrix of non-transformed wet weight of macroalgal species in 5 quadrats for each site. SI = Sant’ Isidoro (Donor); MA = Marittima (Donor); TG = Torre Guaceto (Restoration); PC = Porto Cesareo (Restoration). ** P < 0.01. (DOCX) [file pone.0224477.s003.docx]

**S2 Table**. **Structure of macroalgal assemblage: PERMANOVA and PERMDISP.** Permutational analysis of variance (PERMANOVA) on the structure of macroalgal assemblages in different locations and sites (nested within locations). PERMDISP analysis and pairwise comparisons were used to evaluate the homogeneity of multivariate dispersion among locations. Analyses were done on Bray-Curtis dissimilarity matrix of non-transformed wet weight of macroalgal species in 5 quadrats for each site. SI = Sant’ Isidoro (Donor); MA = Marittima (Donor); TG = Torre Guaceto (Restoration); PC = Porto Cesareo (Restoration). *** P < 0.001

| **Source of variability** | **df** | **MS** | **Pseudo-F** | **Unique perms** |
| --- | --- | --- | --- | --- |
| Location = L | 3 | 4.302 | 9.444 *** | 105 |
| Site (L) | 4 | 0.779 | 1.283 | 9877 |
| Residual | 32 | 4.859 |  |  |
| **PERMDISP** | P (perm) = 0.059 | | | |
|  | F = 2.711, df _1_= 3; df _2_= 36 | | | |
| **Pairwise comparisons** | MA $\neq$SI $\neq$TG $\neq$ PC | | | |
