# Supplementary material for: Are we ready for scaling up restoration actions? An insight from Mediterranean macroalgal canopies
Source: PLoS One. 2019 Oct 25;14(10):e0224477. doi: 10.1371/journal.pone.0224477 (PMC6814225; doi:10.1371/journal.pone.0224477)
Supplement: S3 Table — Similarity percentage analysis (SIMPER) identifying the % contribution of each macroalgal species (g wet weight scraped from 400 cm2 quadrats) to the Bray Curtis dissimilarity metric between pairs of locations. Average dissimilarity: Sant’ Isidoro (SI)—Marittima (MA) = 57.04; Sant’ Isidoro—Torre Guaceto (TG) = 81.64; Marittima—Torre Guaceto = 84.22; Sant’ Isidoro—Porto Cesareo (PC) = 74.19; Marittima—Porto Cesareo = 79.82; Torre Guaceto—Porto Cesareo = 64.86. (DOCX) [file pone.0224477.s004.docx]

**S3 Table. Structure of macroalgal assemblage: SIMPER.**

Similarity percentage analysis (SIMPER) identifying the % contribution of each macroalgal species (g wet weight scraped from 400 cm^2^ quadrats) to the Bray Curtis dissimilarity metric between pairs of locations. Average dissimilarity: Sant’ Isidoro (SI) - Marittima (MA) = 57.04; Sant’ Isidoro - Torre Guaceto (TG) = 81.64; Marittima - Torre Guaceto = 84.22; Sant’ Isidoro - Porto Cesareo (PC) = 74.19; Marittima - Porto Cesareo = 79.82; Torre Guaceto - Porto Cesareo = 64.86.

| **Macroalgal Species** | **% Contr.** | **Cum. %** | **Mean abn.** | **Mean abn.** |
| --- | --- | --- | --- | --- |
|  |  |  | **SI** | **MA** |
| *Jania rubens* | 27.8 | 27.8 | 42.05 | 9.59 |
| *Cystoseira amentacea* | 26.48 | 54.28 | 38.52 | 53.39 |
| *Corallina officinalis* | 7.84 | 62.12 | 3.77 | 9.34 |
| *Titanoderma pustulatum* | 6.04 | 68.16 | 6.16 | 5.22 |
| *Halimeda tuna* | 4.22 | 72.38 | 3.18 | 4.03 |
| *Anadyomene stellata* | 3.1 | 75.48 | 0 | 3.83 |
| *Jania virgata* | 2.54 | 78.02 | 0 | 3.08 |
| *Tenarea tortuosa* | 2.22 | 80.25 | 0 | 2.67 |
| *Padina pavonica* | 2.2 | 82.45 | 1.35 | 1.7 |
| *Amphiroa rigida* | 2.14 | 84.59 | 1.66 | 2.82 |
| *Valonia utricularis* | 1.95 | 86.54 | 2.58 | 1.73 |
| *Cystoseira compressa* | 1.85 | 88.38 | 0 | 2.31 |
| *Gelidiella pannosa* | 1.58 | 89.96 | 0.01 | 1.95 |
| *Laurencia obtusa* | 1.36 | 91.33 | 0.1 | 1.52 |
|  |  |  | **SI** | **TG** |
| *Cystoseira amentacea* | 27.48 | 27.48 | 38.52 | 0 |
| *Jania rubens* | 26.98 | 54.46 | 42.04 | 6.71 |
| *Ellisolandia elongata* | 11.44 | 65.89 | 0.12 | 15.49 |
| *Halimeda tuna* | 5.75 | 71.64 | 3.18 | 7.64 |
| *Laurencia obtusa* | 5.53 | 77.17 | 0.1 | 7.48 |
| *Titanoderma pustulatum* | 5.47 | 82.65 | 6.16 | 7.87 |
| *Corallina officinalis* | 2.93 | 85.57 | 3.77 | 0.87 |
| *Valonia utricularis* | 2.28 | 87.85 | 2.58 | 2.92 |
| *Dictyopteris polypoides* | 1.95 | 89.81 | 0.46 | 2.12 |
| *Anadyomene stellata* | 1.93 | 91.74 | 0 | 2.45 |
|  |  |  | **MA** | **TG** |
| *Cystoseira amentacea* | 36.64 | 36.64 | 53.39 | 0 |
| *Ellisolandia elongata* | 10.53 | 47.16 | 0 | 15.49 |
| *Corallina officinalis* | 6.96 | 54.12 | 9.34 | 0.87 |
| *Jania rubens* | 6.26 | 60.39 | 9.59 | 6.71 |
| *Titanoderma pustulatum* | 5.66 | 66.04 | 5.22 | 7.87 |
| *Laurencia obtusa* | 4.83 | 70.87 | 1.52 | 7.48 |
| *Halimeda tuna* | 4.57 | 75.44 | 4.03 | 7.64 |
| *Anadyomene stellata* | 3.08 | 78.52 | 3.83 | 2.45 |
| *Jania virgata* | 2.13 | 80.65 | 3.08 | 0 |
| *Valonia utricularis* | 2.02 | 82.67 | 1.73 | 2.92 |
| *Amphiroa rigida* | 1.89 | 84.56 | 2.82 | 2.28 |
| *Tenarea tortuosa* | 1.87 | 86.43 | 2.67 | 0 |
| *Cystoseira compressa* | 1.54 | 87.97 | 2.31 | 0 |
| *Dictyopteris polypoides* | 1.51 | 89.48 | 0 | 2.12 |
| *Gelidiella pannosa* | 1.38 | 90.85 | 1.95 | 0.17 |
|  |  |  | **SI** | **PC** |
| *Cystoseira amentacea* | 31.24 | 31.24 | 38.52 | 0 |
| *Jania rubens* | 25 | 56.25 | 42.04 | 14.89 |
| *Halimeda tuna* | 6.78 | 63.03 | 3.18 | 7.34 |
| *Laurencia obtusa* | 5.95 | 68.98 | 0.1 | 7.03 |
| *Titanoderma pustulatum* | 4.53 | 73.51 | 6.16 | 2.33 |
| *Valonia utricularis* | 4.44 | 77.95 | 2.58 | 6.16 |
| *Anadyomene stellata* | 3.78 | 81.73 | 0 | 4.34 |
| *Corallina officinalis* | 3.18 | 84.91 | 3.77 | 0 |
| *Bryopsis corymbosa* | 2.35 | 87.26 | 0.07 | 2.37 |
| *Cystoseira compressa* | 1.87 | 89.13 | 0 | 2.11 |
| *Padina pavonica* | 1.6 | 90.73 | 1.35 | 0.7 |
|  |  |  | **MA** | **PC** |
| *Cystoseira amentacea* | 39.92 | 39.92 | 53.39 | 0 |
| *Jania rubens* | 8.09 | 48.02 | 9.59 | 14.89 |
| *Corallina officinalis* | 7.58 | 55.59 | 9.34 | 0 |
| *Halimeda tuna* | 5.41 | 61 | 4.03 | 7.34 |
| *Laurencia obtusa* | 4.41 | 65.41 | 1.52 | 7.03 |
| *Valonia utricularis* | 4.14 | 69.55 | 1.73 | 6.16 |
| *Titanoderma pustulatum* | 3.84 | 73.39 | 5.22 | 2.33 |
| *Anadyomene stellata* | 3.67 | 77.06 | 3.83 | 4.34 |
| *Cystoseira compressa* | 2.49 | 79.55 | 2.31 | 2.11 |
| *Jania virgata* | 2.32 | 81.87 | 3.08 | 0 |
| *Tenarea tortuosa* | 2.04 | 83.91 | 2.67 | 0 |
| *Bryopsis corymbosa* | 2.01 | 85.92 | 0 | 2.37 |
| *Amphiroa rigida* | 1.98 | 87.9 | 2.82 | 0.69 |
| *Padina pavonica* | 1.76 | 89.66 | 1.7 | 0.7 |
| *Gelidiella pannosa* | 1.49 | 91.15 | 1.95 | 0.15 |
|  |  |  | **TG** | **PC** |
| *Ellisolandia elongata* | 19.72 | 19.72 | 15.49 | 1.4 |
| *Jania rubens* | 14.14 | 33.86 | 6.71 | 14.89 |
| *Halimeda tuna* | 10.27 | 44.13 | 7.64 | 7.34 |
| *Laurencia obtusa* | 9.26 | 53.39 | 7.48 | 7.03 |
| *Titanoderma pustulatum* | 8.39 | 61.78 | 7.87 | 2.33 |
| *Valonia utricolaris* | 7.13 | 68.92 | 2.92 | 6.16 |
| *Anadyomene stellata* | 4.77 | 73.69 | 2.45 | 4.34 |
| *Bryopsis corymbosa* | 3.72 | 77.41 | 0 | 2.37 |
| *Cystoseira compressa* | 2.92 | 80.33 | 0 | 2.11 |
| *Dictyopteris polypoides* | 2.9 | 83.23 | 2.12 | 0 |
| *Amphiroa rigida* | 2.56 | 85.79 | 2.28 | 0.69 |
| *Jania longifurca* | 2.21 | 88 | 1.91 | 0 |
| *Pseudochlorodesmis furcellata* | 1.8 | 89.8 | 1.15 | 0 |
| *Herposiphonia secunda f. tenella* | 1.51 | 91.31 | 0.41 | 1.23 |
