# Supplementary material for: Are we ready for scaling up restoration actions? An insight from Mediterranean macroalgal canopies
Source: PLoS One. 2019 Oct 25;14(10):e0224477. doi: 10.1371/journal.pone.0224477 (PMC6814225; doi:10.1371/journal.pone.0224477)
Supplement: S4 Table — The structure of the random term was selected comparing models with different error structures using the Akaike information criterion (AIC). (DOCX) [file pone.0224477.s005.docx]

**S4 Table. Comparison among linear mixed-effects models assessing the survival of germlings during outplanting**. The structure of the random term was selected comparing models with different error structures using the Akaike information criterion (AIC).

| **Model** | **df** | **AIC** |
| --- | --- | --- |
| Day + Halfday(Day) + (1 \| Halfday) | 5 | 355.35 |
| Day + Halfday(Day) + (1 \| Day) | 5 | 320.30 |
| Day + Halfday(Day) + (1 \| Day/Halfday) | 6 | 316.20 |
| Day + Halfday(Day) + (Halfday \| Day) | 7 | 310.94 |
