# Supplementary material for: Are we ready for scaling up restoration actions? An insight from Mediterranean macroalgal canopies
Source: PLoS One. 2019 Oct 25;14(10):e0224477. doi: 10.1371/journal.pone.0224477 (PMC6814225; doi:10.1371/journal.pone.0224477)
Supplement: S5 Table — Linear mixed-effects model assessing the survival of germlings during outplanting. The number of germlings per tile was log-transformed to normalize the data. Significance of the fixed factors was assessed by mean of the Wald test. (DOCX) [file pone.0224477.s006.docx]

**S5 Table. Factors influencing survival of *C. amentacea* germlings during outplanting.** Linear mixed-effects model assessing the survival of germlings during outplanting. The number of germlings per tile was log-transformed to normalize the data. Significance of the fixed factors was assessed by mean of the Wald test.

| **Fixed effects** | **Estimate** | **Std. Error** | **z** | ***P*** |
| --- | --- | --- | --- | --- |
| Intercept | 8.592 | 0.635 | 13.524 | <0.0001 |
| Day | -1.401 | 0.287 | -4.880 | <0.0001 |
| Halfday (Day) | -0.651 | 0.070 | -9.268 | <0.0001 |
| **Random effects** | **Variance** | **Std. Deviance** | **Corr.** |  |
| Day | 5.488 | 2.343 |  |  |
| Halfday | 0.674 | 0.821 | -1.00 |  |
| Residual | 0.403 | 0.635 |  |  |
