# Supplementary material for: Are we ready for scaling up restoration actions? An insight from Mediterranean macroalgal canopies
Source: PLoS One. 2019 Oct 25;14(10):e0224477. doi: 10.1371/journal.pone.0224477 (PMC6814225; doi:10.1371/journal.pone.0224477)
Supplement: S6 Table — Analysis of variance of the effects of C. amentacea adults and herbivory due to macrograzers on the number of germlings per quadrat survived at different sites and locations after three months. The sum of germlings in the five tiles within each quadrat was log-transformed. Cochran’s C = 0.082. He = free access from macrograzers, No He = cages of grazers exclosure, CA = control of artifact. * P < 0.05. (DOCX) [file pone.0224477.s007.docx]

**S6 Table. Factors influencing survival of *C. amentacea* germlings during early settlement phases.**

Analysis of variance of the effects of *C. amentacea* adults and herbivory due to macrograzers on the number of germlings per quadrat survived at different sites and locations after three months. The sum of germlings in the five tiles within each quadrat was log-transformed. Cochran’s C = 0.219, ns. He = free access from macrograzers, No He = cages of grazers exclosure, CA = control of artifact. * *P* < 0.05

| **Source of variability** | **df** | **MS** | ***F*** |
| --- | --- | --- | --- |
| Location = L | 1 | 6.357 | 39.153 * |
| Adult Transplant = AT | 1 | 5.052 | 5.657 |
| Herbivory = H | 2 | 4.528 | 2.698 |
| Site (Location) = S(L) | 2 | 0.162 | 0.263 |
| L * AT | 1 | 0.678 | 0.759 |
| L * H | 2 | 1.858 | 1.107 |
| AT * H | 2 | 2.741 | 5.215 |
| AT * S(L) | 2 | 0.893 | 1.447 |
| H * S(L) | 4 | 1.678 | 2.718 * |
| L * AT * H | 2 | 0.100 | 0.191 |
| S(L) * AT * H | 4 | 0.526 | 0.852 |
| Residual | 48 | 0.617 |  |
| **Student-Newman-Keuls test** within sites, among levels of herbivory | | | |
| Donor: Marittima Site 1 | He < CA = No He | | |
| Donor: Marittima Site 2 | No He > He; No He = CA; He = CA | | |
| Restoration: T. Guaceto Site 1 | He = CA = No He | | |
| Restoration: T. Guaceto Site 2 | He = CA = No He | | |
