# Supplementary material for: Are we ready for scaling up restoration actions? An insight from Mediterranean macroalgal canopies
Source: PLoS One. 2019 Oct 25;14(10):e0224477. doi: 10.1371/journal.pone.0224477 (PMC6814225; doi:10.1371/journal.pone.0224477)
Supplement: S7 Table — Analysis of variance on the abundance of C. amentacea adults (cover / 400 cm2 quadrats) among different herbivory treatments (free access from macrograzers, grazers exclosure, control of artifact) in different conditions (transplanted at restoration sites and unmanipulated at donor sites), at different locations (two levels, nested within condition) and sites (two levels, nested within location). Cochran’s C = 0.185. = 0.191 ** P < 0.01. (DOCX) [file pone.0224477.s008.docx]

**S7 Table. Efficacy of *C. amentacea* adult transplant: ANOVA.** Analysis of variance on the abundance of *C. amentacea* adults (cover / 400 cm^2^ quadrats) among different herbivory treatments (free access from macrograzers, grazers exclosure, control of artifact) in different conditions (transplanted at restoration sites and unmanipulated at donor sites), at different locations (two levels, nested within condition) and sites (two levels, nested within location). Cochran’s C = 0.185. = 0.191 ** *P* < 0.01

| **Source of variability** | **df** | **MS** | **F** |
| --- | --- | --- | --- |
| Condition = C | 1 | 4512.5 | 2.397 |
| Location (Condition) = L | 2 | 1882.3 | 6.164 |
| Herbivory = H | 2 | 372.9 | 0.948 |
| Site (Location (Condition)) = S(L(C)) | 4 | 305.4 | 4.095 ** |
| H * C | 2 | 372.9 | 2.964 |
| H * L(C) | 4 | 125.8 | 2.154 |
| H * S(L(C)) | 8 | 58.4 | 0.783 |
| Residual | 48 | 74.6 |  |
| **Student-Newman-Keuls test** between sites | | | |
| Marittima | Site 1 = Site 2 | | |
| Sant’Isidoro | Site 1 < Site 2, ** | | |
| Torre Guaceto | Site 1 = Site 2 | | |
| Porto Cesareo | Site 1 = Site 2 | | |
