# Supplementary figures and images for: Are we ready for scaling up restoration actions? An insight from Mediterranean macroalgal canopies
Source: PLoS One. 2019 Oct 25;14(10):e0224477. doi: 10.1371/journal.pone.0224477 (PMC6814225; doi:10.1371/journal.pone.0224477)

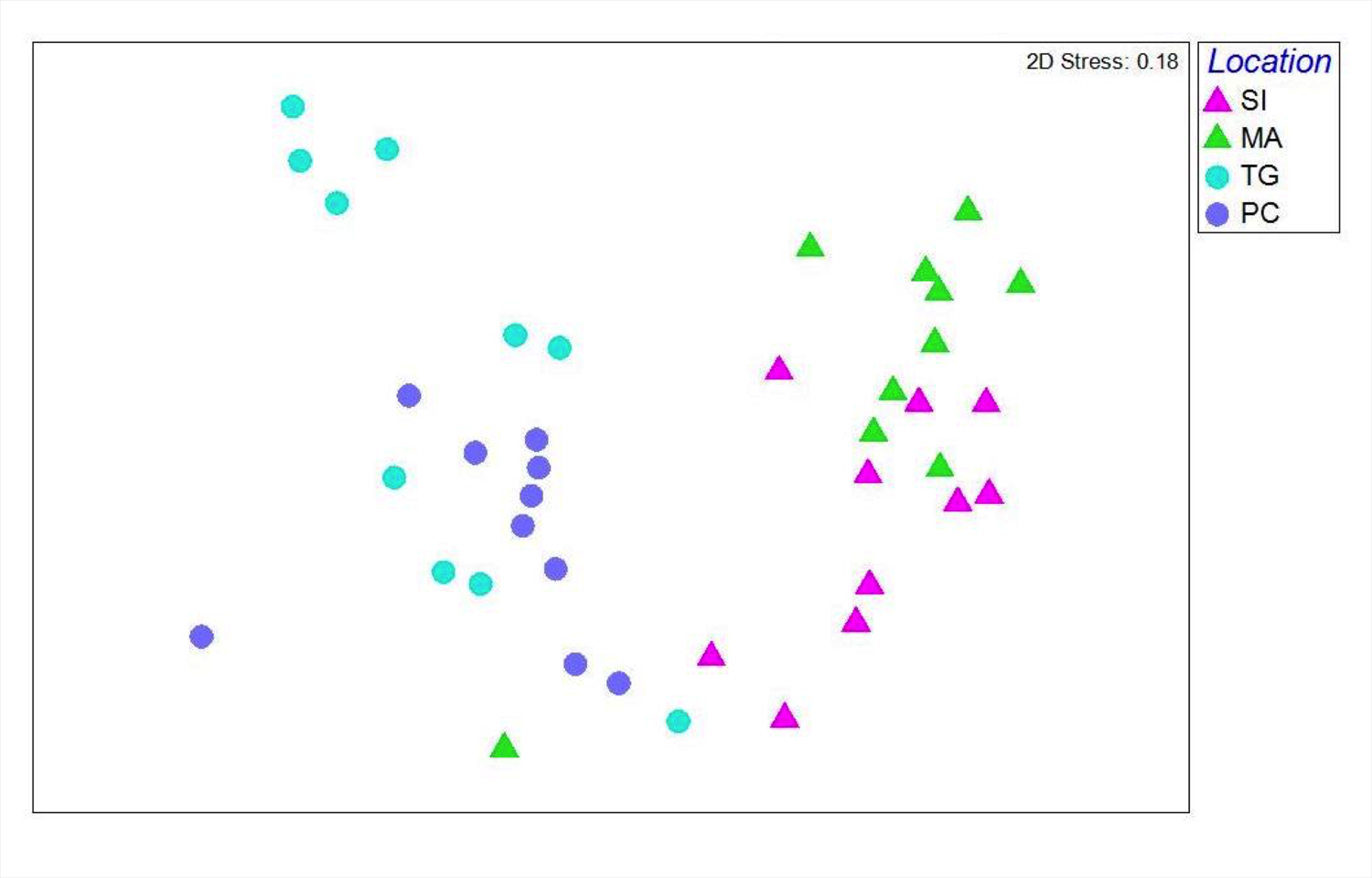

Supplement: S1 Fig — Multidimensional scaling (MDS) of the structure of macroalgal assemblage at experimental locations. SI = Sant’Isidoro (Donor); MA = Marittima (Donor); TG = Torre Guaceto (Restoration); PC = Porto Cesareo (Restoration). (TIFF) [file pone.0224477.s009.tiff]

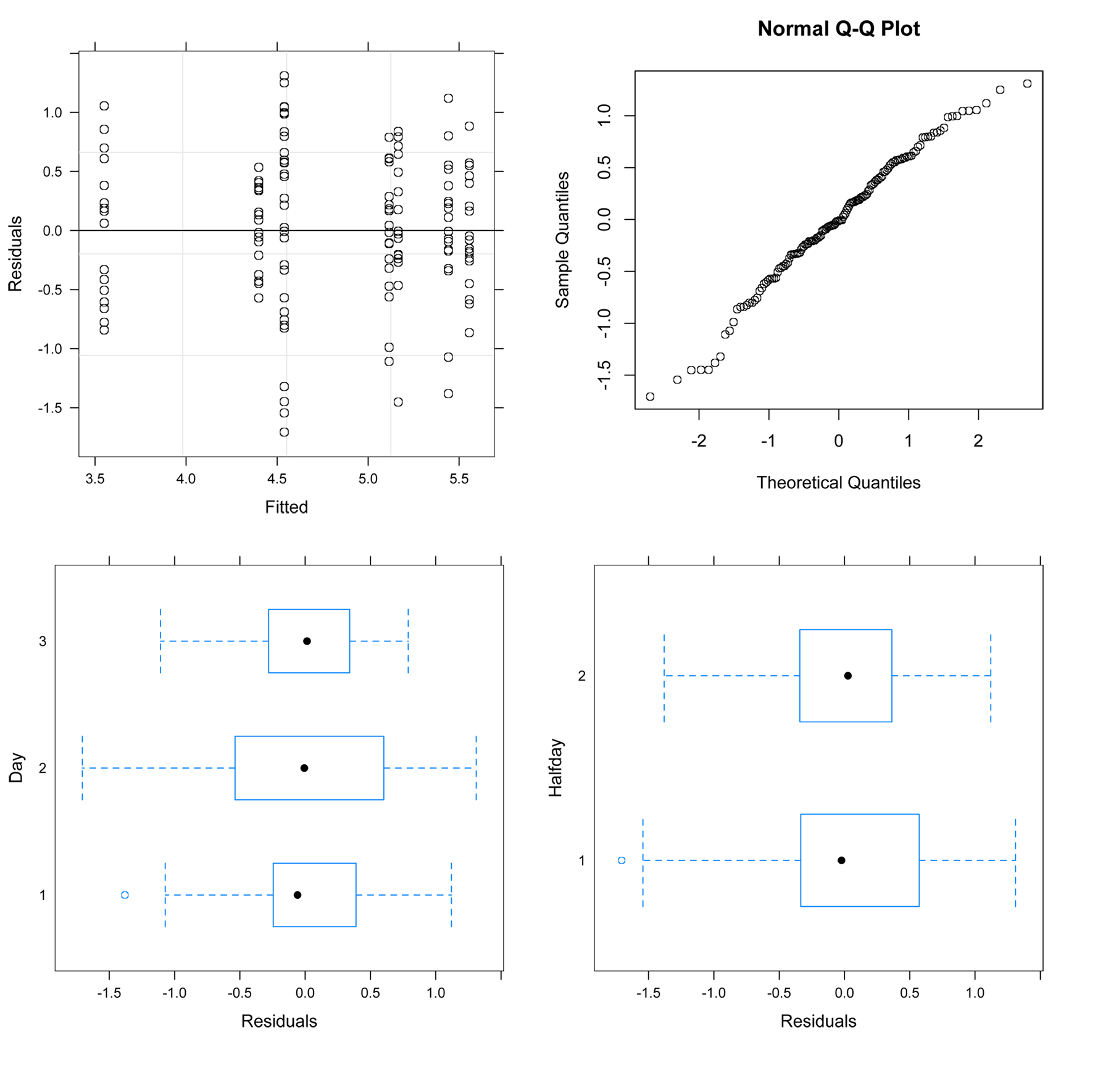

Supplement: S2 Fig — Plot of the residuals showing the linearity and normality of the data after the logarithmic transformation of the number of germlings per tile. (TIF) [file pone.0224477.s010.tif]

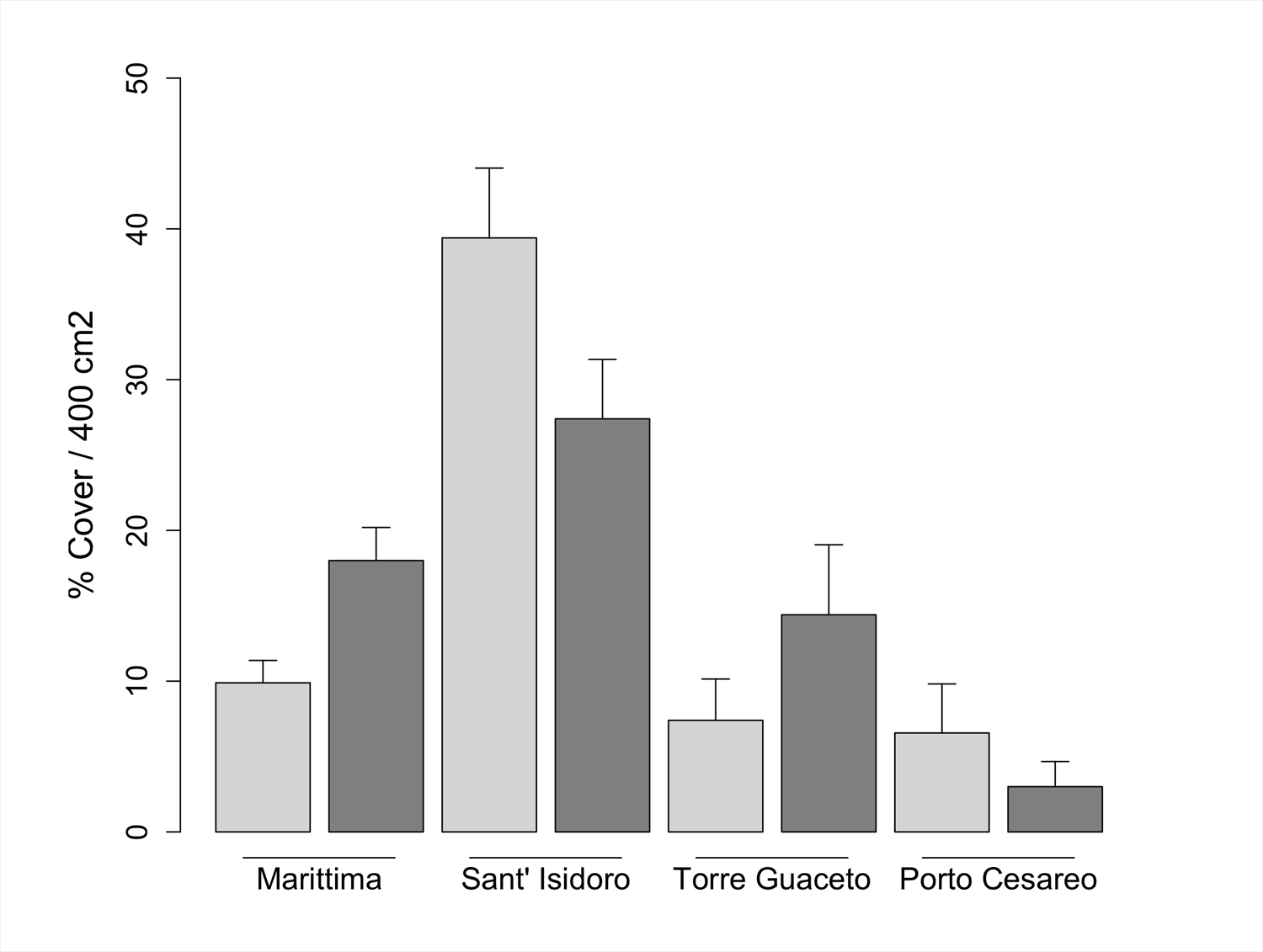

Supplement: S3 Fig — Cover of C. amentacea adults at experimental sites in October 2017. Light grey = site 1; dark grey = site 2 within each location, N = 144. (TIFF) [file pone.0224477.s011.tiff]
